# CXCL12 overexpression promotes the angiogenesis potential of periodontal ligament stem cells

**DOI:** 10.1038/s41598-017-10971-1

**Published:** 2017-08-31

**Authors:** Lei Zhang, Yong Zhou, Xiaoyu Sun, Jian Zhou, Pishan Yang

**Affiliations:** 1 Department of Periodontology, School of Stomatology, Shandong University, Jinan, Shandong 250012 China; 2 Department of Periodontology, College and Hospital of Stomatology, Anhui Medical University, Hefei, 230032 Anhui China; 3 Shandong Provincial Key Laboratory of Oral Biomedicine, Shandong University, Jinan, Shandong 250012 China

## Abstract

Periodontal ligament stem cells (PDLSCs) are a major source of mesenchymal stem cells (MSCs) in adults and are effective for tissue engineering, like promoting angiogenesis and bone regeneration. CXCL12 has been reported to be involved in the recruitment and engraftment of MSCs in wound sites. However, whether CXCL12 potentiates the angiogenesis of PDLSCs is not clear. In this experiment, we transduced PDLSCs with CXCL12, and evaluated the angiogenesis potential of CXCL12-modified PDLSCs through *in vitro* and *in vivo* studies. The results showed that CXCL12 overexpression significantly stimulated the gene and protein expressions of bFGF, VEGF, SCF and PLGF in PDLSCs; CXCL12 gene modified PDLSCs formed longer capillary‐like structure; Moreover, *in vivo* transplanted PDLSCs transduced with CXCL12 could significantly promote bone tissue repair and angiogenesis in a rat critical-sized calvarial bone defect model. Taken together, our study confirmed that CXCL12 can enhance the angiogenesis potential of PDLSCs, which are crucial in the repair and regeneration of bone tissue.

## Introduction

Bone fractures, malformations, surgical removal of tumors and periodontitis can cause bone defects that interfere with normal bone function and configuration and have become serious clinical problems. Regeneration of damaged bone tissue has been a major goal for both clinicians and researchers worldwide. Currently, autologous and allogenic bone grafts are the primary treatment modalities. However, clinical application of these bone grafts is limited due to the high cost of bone-harvesting procedures and are associated with donor site morbidity, inflammation, and pain^1, 2^.

Bone tissue engineering provides several advantages, including high-quality regeneration of damaged tissues, no donor site harvesting, and low risk of disease transmission or autoimmune rejection^3^. Studies have shown that dental-derived mesenchymal stem cells (MSCs) may have superior differentiation capacity compared with human bone marrow mesenchymal stem cells (hBMMSCs)^4–7^. Among the different types of dental MSCs that have been identified thus far, stem cells from periodontal ligaments (PDLSCs) are of special interest. Studies have confirmed the multilineage differentiation capacity of PDLSCs *in vitro* and *in vivo*, which suggests that PDLSCs are ideal cell sources for cell-based periodontal/bone regeneration therapy^8–10^.

In addition to directly differentiating into bone- forming cells, MSCs also enhance blood vessel regeneration and vascularization, which is crucial for bone regeneration and survival of newly formed bone tissues. CXCL12 (also known as SDF-1) is a chemokine that regulates bone marrow-derived mesenchymal stem/stromal cells (BMSCs) migration by interacting with CXCR4^11, 12^. However, recent studies have also shown that the CXCL12 not only regulates cell migration but also promotes angiogenesis in damaged tissue^13, 14^. CXCL12 has been reported to induce neovascularization in ischemic lesion, tumor, and wounded tissues^15–17^. In addition, CXCL12 acts synergistically with vascular endothelial growth factor (VEGF) to enhance angiogenesis^18, 19^. Therefore, it is reasonable to hypothesize that CXCL12 overexpression can potentiate PDLSC angiogenesis.

Here, to test our hypothesis, we investigated whether CXCL12 can enhance PDLSC-mediated angiogenesis and bone tissue repair through *in vitro* and *in vivo* studies.

## Results

### Mesenchymal stem cell markers expression and multilineage differentiation potential of PDLSCs

The flow cytometry analysis (Supporting Fig. S1A) showed that PDLSCs expressed mesenchymal stem cells markers, like CD90, CD44 and CD105. CD45 was not detected.

The results of Oil Red O (Supporting Fig. S1B) and alkaline phosphatase (Supporting Fig. S1C) staining showed that PDLSCs possessed multilineage differentiation potential.

### CXCL12 overexpression enhances angiogenesis of PDLSCs *in vitro*

**Figure 1:**
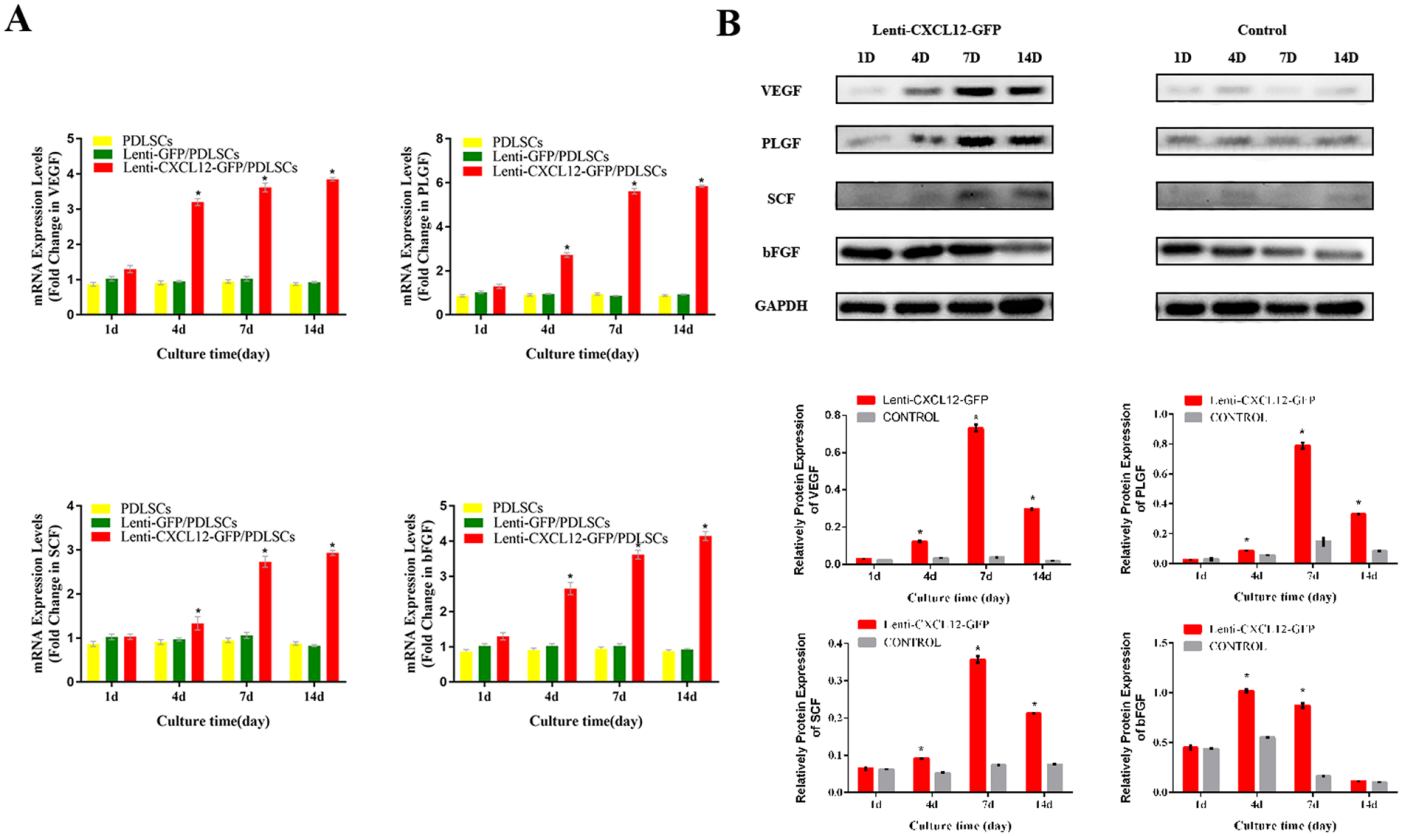
Quantitative RT-qPCR and Western-blot analysis of angiogenesis-associated factors in CXCL12 modified PDLSCs. PDLSCs were transduced with Lenti-CXCL12-GFP and Lenti-GFP and mRNA (**A**) and protein (**B**) levels of angiogenesis-associated factors were measured by RT-qPCR and Western blot assays respectively at post-transduction day 1, 4, 7 and 14. All experiments were performed three times (N = 3). *p < 0.05.

To assess the effect of CXCL12 overexpression on promoting angiogenesis potential, PDLSCs were transduced with Lenti-CXCL12-GFP and Lenti-GFP. The mRNA and protein levels of angiogenesis-associated factors were measured at post-transduction day 1, 4, 7, and 14. The mRNA expression of VEGF, which involves the regulation of blood vessel formation, was significantly increased on post-transduction day 4 in CXCL12-transduced PDLSCs and continued to increase at day 7 and day 14 compared with GFP control group (Fig. 1A). The expression level of other critical angiogenic genes, such as basic fibroblast growth factor (bFGF), placental growth factor (PLGF) and stem cell factor (SCF), showed a general tendency identical to that of VEGF. The protein expression of angiogenic markers in gene modified PDLSCs exhibited the same trend as that observed with qPCR. The expressions of VEGF, SCF bFGF and PLGF protein increased obviously from day 7 in Lenti-CXCL12-transduced group compared with control (Fig. 1B). These data indicated that CXCL12 overexpression potentiate mRNA and protein expression of angiogenic markers in PDLSCs *in vitro*.

**Figure 2:**
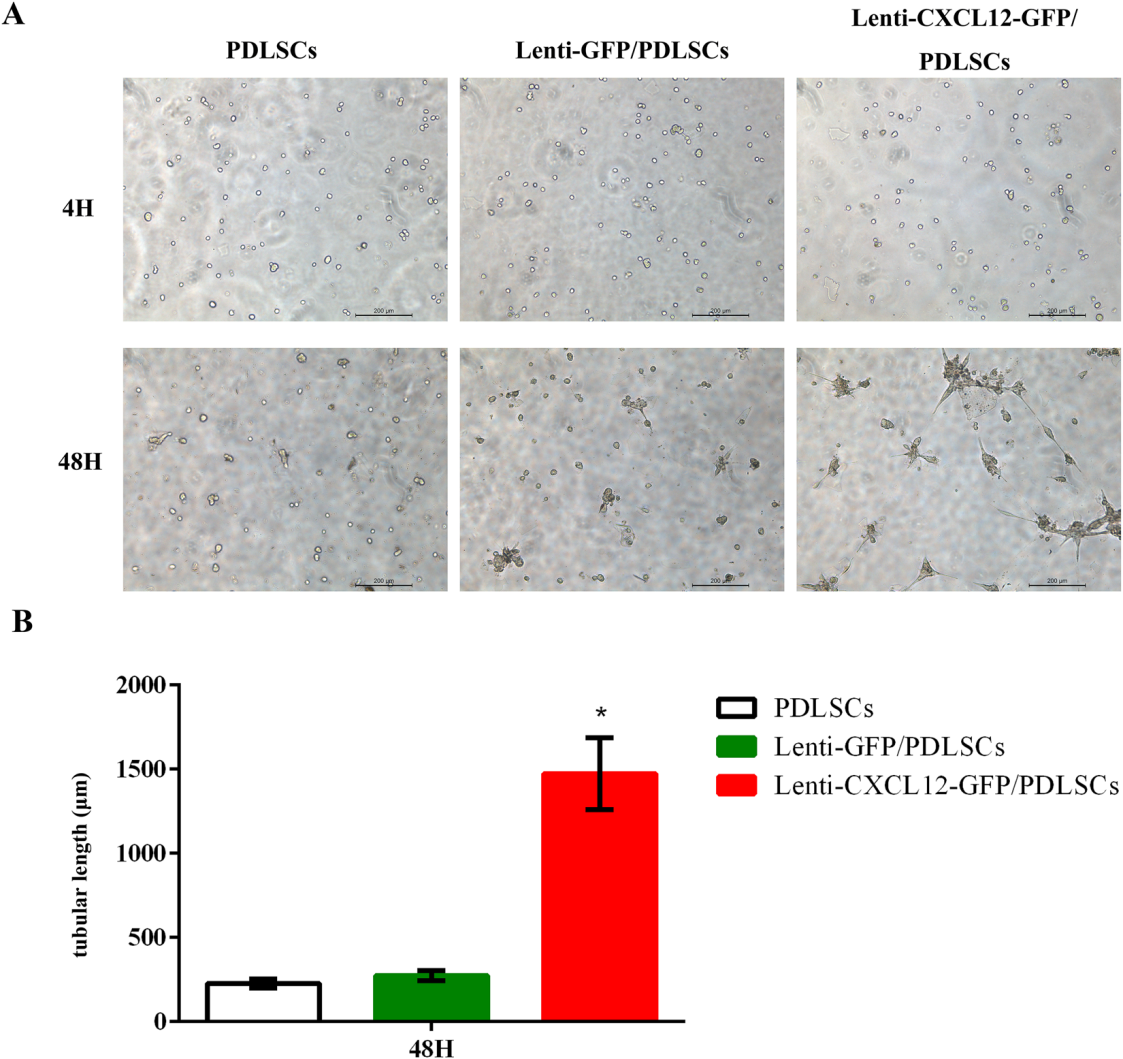
The vessel-like structure formation by CXCL12 modified PDLSCs on the surface of Matrigel. PDLSCs, Lenti-GFP/PDLSCs and Lenti-CXCL12-GFP/PDLSCs were seeded (2 × 10^4^ cells/well) in 96-well plates coated with Matrigel and images were captured at 4 h and 48 h (**A**); statistical analysis showed longer vessel-like structure formation in Lenti-CXCL12-GFP/PDLSCs than in PDLSCs and Lenti-GFP/PDLSCs (**B**). *p < 0.05.

To further investigate whether CXCL12-modified PDLSCs possess angiogenic function, the *in vitro* Matrigel angiogenesis assay was performed. Lenti-GFP/PDLSCs, Lenti-CXCL12-GFP/PDLSCs, and PDLSCs were seeded onto Matrigel, and vascular-like structures were evaluated. The resulting vessel-like structures were shown in Fig. 2. Image analysis demonstrated a significant increase in tubule length in the Lenti-CXCL12-GFP/PDLSCs at 48 h compared with the other control groups, which quantitatively demonstrated the angiogenesis-facilitating effect.

### CXCL12 promoted PDLSCs bone regeneration and angiogenic potential in a rat critical-sized calvarial defect model

**Figure 3:**
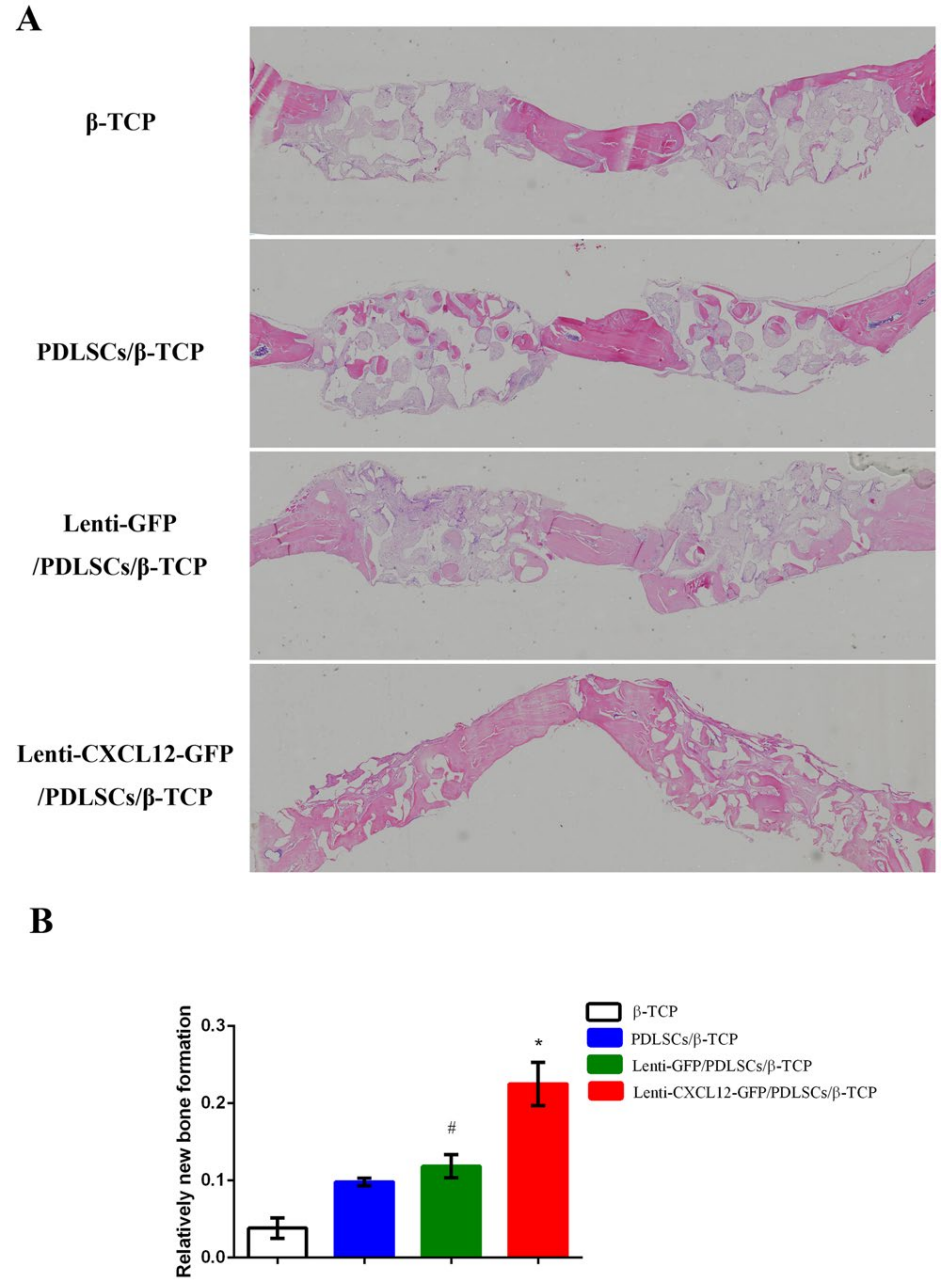
The bone regeneration potential of CXCL12 modified PDLSCs in a rat critical-sized calvarial defects model. Lenti-CXCL12-GFP/PDLSCs/β-TCP, Lenti-GFP/PDLSCs/β-TCP, PDLSCs/β-TCP and β-TCP were respectively grafted into rat critical-sized calvarial defects for 8 weeks. HE staining for specimens of bone defects was conducted (**A**) and a new bone formation percentage accounted (**B**). *p < 0.05, ^#^ *p* < 0.05.

Two bilateral 5 mm diameter critical-sized calvarial defects were created in rats and filled with β-TCP, Lenti-CXCL12-GFP/PDLSCs/β-TCP, Lenti-GFP/PDLSCs/β-TCP group, PDLSCs/β-TCP group respectively. Eight weeks later, the rats were sacrificed and specimens of bone defects examined by histology and immunohistochemistry. The results of hematoxylin and eosin (HE) staining of decalcified specimens showed that the percentage of new bone formation area was significantly higher in the Lenti-GFP/PDLSCs/β-TCP group (0.118 ± 0.013) than that in the β-TCP group (0.038 ± 0.014), while compared with that in the Lenti-GFP/PDLSCs/β-TCP group, 2 times more new bone could be detected in the Lenti-CXCL12-GFP/PDLSCs/β-TCP group (0.225 ± 0.021). By contrast, there was no significant difference between the β- Lenti-GFP/PDLSCs/β-TCP group and PDLSCs/β-TCP group (Fig. 3).

**Figure 4:**
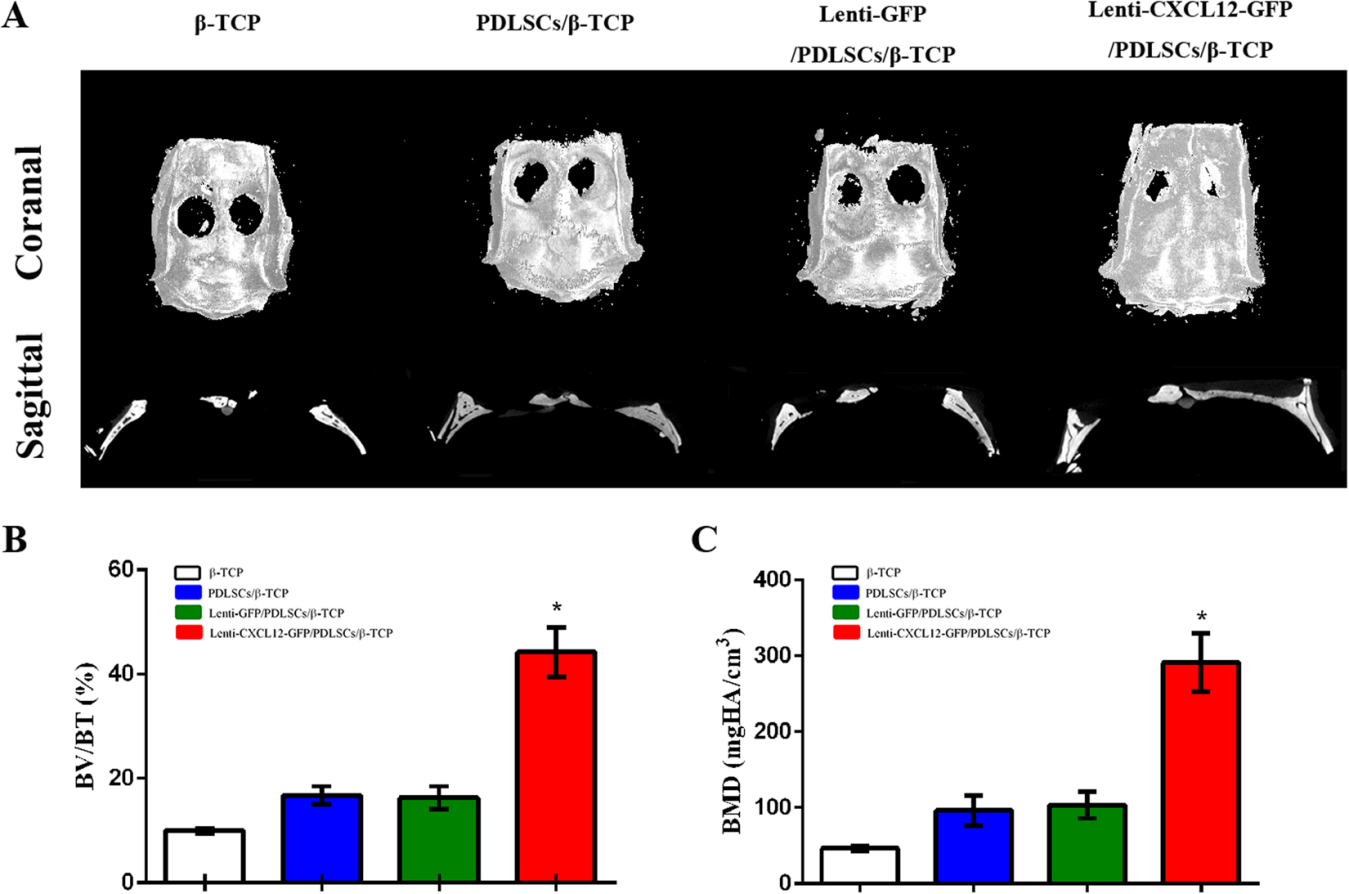
Micro-CT evaluation of the repaired skull at 8 weeks after implantation. β-TCP group, PDLSCs/β-TCP group, Lenti-GFP/PDLSCs/β-TCP group and Lenti-CXCL12-GFP/PDLSCs/β-TCP group. (from left to right). Micro-CT images of calvarial defects taken 8 weeks after implantation (**A**). Morphometric analysis of new bone formation (**B**) and local bone mineral density (**C**). *p < 0.05.

Morphology of the newly formed bone was also detected by micro-CT. Substantial formation of plate-like bone structures was visible in the defect site 8 weeks after treatment with target gene-transduced PDLSCs. The new bone had expanded and occupied almost the whole region of the bone defect observed by coronal and sagittal surface (Fig. 4A). Quantitative analysis of the newly formed bone was performed using morphometrical methods. This analysis confirmed that more bone formation occurred in the target gene groups (*p < 0.05). The ratio of new BV to total BV (BV/TV, %), which indicates the relative amount of newly formed bone, was 45.68% in the Lenti-CXCL12-GFP/PDLSCs/β-TCP group, 18.45% in the Lenti- GFP/PDLSCs/β-TCP group, 17.29% in the PDLSCs/β-TCP group, and 9.52% in theβ-TCP group (Fig. 4B). Local BMDs demonstrated the same tendencies that bone formation was more in the target gene groups than the control group (Fig. 4C).

**Figure 5:**
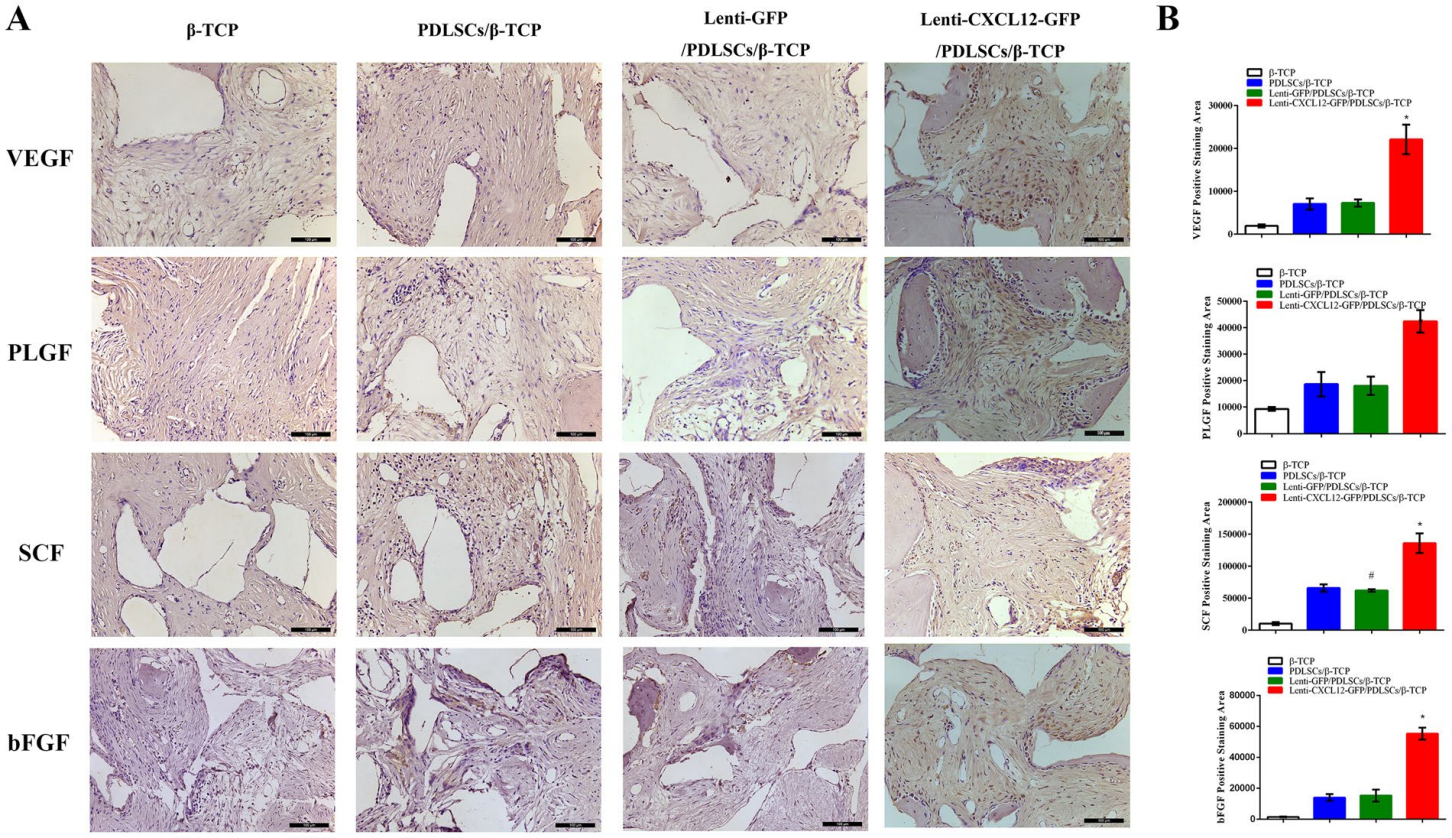
The angiogenesis potential of CXCL12 modified PDLSCs in a rat critical-sized calvarial defects model. Lenti-CXCL12-GFP/PDLSCs/β-TCP, Lenti-GFP/PDLSCs/β-TCP, PDLSCs/β-TCP and β-TCP were respectively grafted into rat critical-sized calvarial defects for 8 weeks and expressions of angiogenic markers in specimens of bone defects were examined with IHC (**A**) and quantitatively analyzed (**B**). All experiments were performed six times (N = 6). *p < 0.05, ^#^ *p* < 0.05.

Similar trends can be observed in the immunohistochemistry assays. It is revealed that there were stronger expressions of VEGF, PLGF, bFGF and SCF in Lenti-CXCL12-GFP/PDLSCs/β-TCP group than that in the other three groups (Fig. 5).

## Discussion

Previous studies have reported on the many disadvantages associated with current bone regeneration treatment modalities in craniofacial reconstructive surgery and regenerative medicine^20–22^. We demonstrate here that CXCL12 can enhance the expression of several growth factors associated with angiogenesis when transduced into PDLSCs. This approach shows promise as a therapeutic option and does not suffer from the drawbacks of current bone regeneration techniques^23–25^. Periodontal ligament tissue provides a superior source of MSCs owing to their accessibility and availability for autologous transplantation^7, 23^. Using human PDLSCs for tissue regeneration might be a promising cellular-based modality for bone tissue engineering. However, according to the results of the current study, transplanted PDLSCs only result in mild bone regeneration capacity.

CXCL12 has been extensively studied, including its roles in migration, homing, proliferation, and angiogenesis^11, 16, 26–29^. Many studies have shown that BMSCs can migrate to injured tissues via the CXCL12/CXCR4 axis and relieve myocardial infarction^11, 30^, promote wound healing^17^, heal bone fracture^31^, and reduce cerebral ischemia^32^. CXCL12 expression is upregulated by hypoxia-inducible factor-1 (HIF-1)^33^, and the CXCL12/CXCR4 axis has been shown to stimulate BMSCs to express VEGF, ANG-1, HGF, and TGF-β, promoting further angiogenesis *in vitro*. VEGF, a potent inducer of angiogenesis, promotes the repair of injured vascular endothelial cells and neovascularization. Studies have shown that CXCL12 promotes the synthesis and secretion of VEGF, and CXCL12 combined with VEGF enhances ischemic angiogenesis^15, 18, 34^.

In our study, we demonstrate that CXCL12 can enhance the expression of VEGF, PLGF, bFGF and SCF genes *in vitro*. Similar trends were also observed in western blotting and Matrigel experiments. To determine whether CXCL12 improves bone regeneration and plays an important role in angiogenesis *in vivo*, PDLSCs were transplanted into critical-sized calvarial bone defects. We found that CXCL12 induced angiogenesis and the expression of VEGF, PLGF, bFGF and SCF in the regenerated bone was higher in the Lenti-CXCL12-GFP/PDLSCs/β-TCP group than in the control groups. Taken together, the results showed that transplanted PDLSCs transduced with CXCL12 repaired the damaged bone tissue by secreting a large number of cellular growth factors.

CXCL12 levels have been reported to be increased at both the mRNA and protein levels during bone graft repair, and anti-SDF-1 (CXCL12) antibody inhibited new bone formation^35^. Moreover, Jones *et al*. demonstrated in a murine osteogenesis imperfecta model that MSC migration to bone and bone marrow is CXCL12-dependent and that CXCL12 upregulates CXCR4, demonstrating chemotaxis *in vitro* and enhancing engraftment *in vivo* ^36^. Inflammation and hypoxia play an important role in regulating the SDF-1/CXCR4 axis. Therefore, we will further investigate whether the effect of CXCL12 on the induction of BMSC migration contributes to the results obtained from the *in vivo* experiment.

## Methods

### PDLSCs isolation and culture

Human PDLSCs were isolated and cultured according to previously published protocols by Seo *et al*.^5^ and Zhang *et al*.^7^. Teeth and periodontal ligament tissues were obtained from healthy male patients (18–25 years old) without any history of periodontal disease who were undergoing third molar extractions. Written informed consent was provided by all patients, and ethical approval was obtained from the Ethics Committee of the School of Stomatology, Shandong University. Briefly, periodontal ligament tissues were gently separated from the surface of the root and digested in the solution of collagenase type I (Worthington Biochem) and dispase (Roche) for 1 h at 37 °C. Single-cell suspensions were obtained by passing the cells through a 70 µm filter. To identify the PDLSCs, single-cell suspensions (1 × 10^4^ cells) were seeded into 10-cm culture dishes (Costar) with α minimum essential medium (αMEM) (Gibco BRL) supplemented with 10% fetal calf serum (Gibco, Inc.), 100 mM ascorbic acid 2-phosphate (Sigma-Aldrich), 2 mM glutamine, 100 U/mL penicillin, and 100 mg/mL streptomycin (Sigma-Aldrich) and then incubated at 37 °C in 5% CO_2_.

### PDLSCs Multi-differentiation

PDLSCs were cultured in DMEM (Sigma-Aldrich, St. Louis, MO) supplemented with 10% fetal bovine serum (FBS), and 1% penicillin/streptomycin. To induce osteoblast differentiation, primary UCBMSCs were grown to confluence in 6-well plates, and the medium was supplemented with calcifying agents (vitamin C, beta-glycerophosphate, and dexamethasone). Cells cultured for up to 28 days were used to assess biomineralization using alkaline phosphatase (ALP) staining (Sigma-Aldrich). For adipogenesis, a specific adipogenesis medium was used (Cyagen, China) and oil red O staining were used to detect the result.

### Flow cytometry

PDLSC were harvested and fixed in 4% paraformaldehyde-phosphate buffer for 20 min. Blocking was performed in PBS containing 2% bovine serum albumin and 0.02% Triton-X 100. After washing with 2% fetal bovine serum-PBS, 2 × 10^5^ cells were stained with the following antibodies for 60 min. Mouse anti-human PE-conjugated CD146 and CD105 antibody (BD Bioscience CA); Mouse anti-human APC-conjugated CD90 antibody (BD Bioscience CA);Mouse anti-human FITC-conjugated CD44 and CD45 (1:10; BD Bioscience); All flow cytometry tests were performed on a FACS Aria (BD Bioscience).

### Cell Transduction

PDLSCs were passaged until the third generation and then transduced with a lentivirus. The control vector was a replication-defective lentivirus encoding GFP (Lenti-GFP). Large-scale lentiviral production was performed by Shanghai R&S Biotechnology Co., Ltd. (Shanghai, China). The efficiency of lentiviral gene transfer in PDLSCs was greatest when the multiplicity of infection (MOI) was 8. The transduction efficiency was assessed by determining the fluorescence intensity after 4 d of culture.

### Real‐time PCR Assay

For RT-qPCR analysis, total RNA was extracted using TRIzol (Sigma-Aldrich, USA). cDNA synthesis was performed using a PrimeScript RT kit following the manufacturer’s instructions (Takara, Japan). RT-qPCR was performed using SYBR premix Ex Taq (Takara, Japan), and ROX fluorescence was then analyzed using a Stratagene Mx3000p system (Agilent Technologies, USA).

### Western‐Blot Assay

Western blot assays were performed according to a previously published protocol^37^. Briefly, cells were harvested before boiling in sample loading buffer for 10 min. Proteins were resolved by 12% sodium dodecyl sulfate polyacrylamide gel electrophoresis (SDS-PAGE); after electrophoresis, the proteins were transferred to a PVDF membrane (Millipore, USA) and subjected to immunoblot analysis using the indicated antibodies. Proteins were visualized using an enhanced chemiluminescence method.

### Capillary‐like Formation Assay

The third passage of PDLSCs, Lenti-GFP/PDLSCs and Lenti-CXCL12-GFP/PDLSCs were seeded (2 × 10^4^ cells/well) in 96-well plates coated with Matrigel (BD Biosciences, USA). Images were captured at the different time points of the experiment.

### Surgical craniotomies

The experimental protocols were approved by the Animal Welfare Committee of Shandong University. Classical porous β-TCP scaffolds (5 mm diameter and 2 mm depth) with an average pore size of 500 μm and 75% porosity were used as exosome carriers. Aliquots of 1 × 10^6^/mL PDLSCs were dropped onto each β-TCP scaffold under sterile conditions for at least 4 h.

Surgical procedures were performed as previously described. Briefly, 24 rats were anesthetized, a 1.5-cm sagittal incision was made in the scalp, and the calvarium was exposed by blunt dissection. Two bilateral 5-mm-diameter critical-sized calvarial defects were created using a dental trephine, and then scaffolds were implanted into the defects. The rats were randomly divided into four groups: β-TCP only group, Lenti-CXCL12-GFP/PDLSCs/β-TCP group, Lenti-GFP/PDLSCs/β-TCP group, and PDLSCs/β-TCP group. There were 6 rats in each groups (N = 6). Eight weeks after surgery, the rats were sacrificed, and crania were harvested and fixed in a 4% paraformaldehyde solution buffered with 0.1 M phosphate solution (pH 7.2) before further analysis.

### Micro-CT Measurement

The specimens were harvested at 8 weeks postoperatively, and bone volume (BV) in the skull was assessed using a desktop microtomographic imaging system (MicroCT-80, Scanco Medical, Bassersdorf, Switzerland). Three-dimensional isosurface renderings were made for the visualization of bone with software. To obtain parameters of BV fraction and bone mineral densities (BMDs) in the defect areas, the gray-values of the voxels were stratified in a histogram running from 225 to 500 with a range of 25. Micro-CT measurements included BV to total bone volume (BV/TV) and local BMDs in the bone defect.

### Histological Examination

The specimens were fixed in 10% formalin and embedded in paraffin, and 5-μm-thick sections were obtained. HE staining and immunohistochemical examination was performed.

### Statistical Analyses

Statistics were presented as the mean ± s.d. An ANOVA with Tukey’s post hoc test was used to assess statistical significance. Differences were considered significant at p-values of <0.05 (*p < 0.05, when the lenti-CXCL12-GFP groups were compared with the Lenti-GFP group; ^#^p < 0.05, when the Lenti-GFP group was compared with the Control groups).

## Electronic supplementary material

SUPPLEMENTARY INFO

## References

[1] Sachlos E, Czernuszka JT (2003). Making tissue engineering scaffolds work. Review on the application of solid freeform fabrication technology to the production of tissue engineering scaffold. Eur. Cell. Mater..

[2] Chen FM (2010). A review on endogenous regenerative technology in periodontal regenerative medicine. Biomaterials..

[3] Monaco E, Bionaz M, Hollister SJ, Wheeler MB (2011). Strategies for regeneration of the bone using porcine adult adiposederived mesenchymal stem cells. Theriogenology..

[4] Gronthos S, Mankani M, Brahim J, Robey PG, Shi S (2010). Postnatal human dental pulp stem cells (DPSCs) *in vitro* and *in vivo*. Proc. Natl. Acad. Sci. USA.

[5] Seo BM (2004). Investigation of multipotent postnatal stem cells from human periodontal ligament. Lancet..

[6] Gronthos S (2002). Stem cell properties of human dental pulp stem cells. J. Dent. Res..

[7] Zhang Q (2009). Mesenchymal stem cells derived from human gingiva are capable of immunomodulatory functions and ameliorate inflammation-related tissue destruction in experimental colitis. J. Immunol..

[8] Iwata T (2010). Validation of human periodontal ligament derived cells as a reliable source for cytotherapeutic use. J. Clin. Periodontol..

[9] Mitrano TI (2010). Culture and characterization of mesenchymal stem cells from human gingival tissue. J. Periodontol..

[10] Tomar GB (2010). Human gingiva-derived mesenchymal stem cells are superior to bone marrow derived mesenchymal stem cells for cell therapy in regenerative medicine. Biochem. Biophys. Res. Commun..

[11] Yu J (2010). SDF-1/CXCR4-mediated migration of transplanted bone marrow stromal cells toward areas of heart myocardial infarction through activation of PI3K/Akt. J. Cardiovasc. Pharmacol..

[12] Theiss HD (2011). Dual stem cell therapy after myocardial infarction acts specifically by enhanced homing via the SDF-1/CXCR4 axis. Stem Cell Res..

[13] Salcedo R (1999). Vascular endothelial growth factor and basic fibroblast growth factor induce expression of CXCR4 on human endothelial cells. *In vivo* neovascularization induced by stromal-derived factor-1alpha. Am. J. Pathol..

[14] Du L, Yang P, Ge S (2012). Stromal cell-derived factor-1 significantly induces proliferation, migration, and collagen type i expression in a human periodontal ligament stem cell subpopulation. J. Periodontol..

[15] Hiasa K (2004). Gene transfer of stromal cell-derived factor-1𝛼 enhances ischemic vasculogenesis and angiogenesis via vascular endothelial growth factor/endothelial nitric oxide synthase-related pathway: next generation chemokine therapy for therapeutic neovascularization. Circulation..

[16] Mukherjee D, Zhao J (2013). The role of chemokine receptor CXCR4 in breast cancer metastasis. Am. J. Cancer Res..

[17] Xu X (2013). Stromal cell-derived factor-1 enhances wound healing through recruiting bone marrow derived mesenchymal stem cells to the wound area and promoting neovascularization. Cells Tissues Organs..

[18] Yu JX (2009). Combination of stromal derived factor-1𝛼 and vascular endothelial growth factor gene modified endothelial progenitor cells is more effective for ischemic neovascularization. J. Vasc. Surg..

[19] Tang JM (2011). VEGF/SDF-1 promotes cardiac stem cell mobilization and myocardial repair in the infarcted heart. Cardiovas. Res..

[20] Mauney JR (2006). Matrix-mediated retention of *in vitro* osteogenic differentiation potential and *in vivo* bone-forming capacity by human adult bone marrow derived mesenchymal stem cells during *ex vivo* expansion. J. Biomed. Mater. Res. A..

[21] Bidarra SJ, Barrias CC, Barbosa MA, Soares R, Granja PL (2010). Immobilization of human mesenchymal stem cells within RGD-grafted alginate microspheres and assessment of their angiogenic potential. Biomacromolecules..

[22] Pham QP (2008). The influence of an *in vitro* generated bone-like extracellular matrix on osteoblastic gene expression of marrow stromal cells. Biomaterials..

[23] Seo BM (2008). SHED repair critical-size calvarial defects in mice. Oral Dis..

[24] Cowan CM (2004). Adipose-derived adult stromal cells heal critical-size mouse calvarial defects. Nat. Biotechnol..

[25] Liu Y (2011). Mesenchymal stem cell-based tissue regeneration is governed by recipient T lymphocytes via IFN-g and TNF-a. Nat. Med..

[26] Gong J (2014). The SDF-1/CXCR4 axis regulates migration of transplanted bone marrow mesenchymal stem cells towards the pancreas in rats with acute pancreatitis. Mol. Med. Rep..

[27] Theiss HD (2011). Dual stem cell therapy after myocardial infarction acts specifically by enhanced homing via the SDF-1/CXCR4 axis. Stem Cell Res..

[28] Salcedo R (1999). Vascular endothelial growth factor and basic fibroblast growth factor induce expression of CXCR4 on human endothelial cells. *In vivo* neovascularization induced by stromal-derived factor-1alpha. Am. J. Pathol..

[29] Burger JA, Kipps TJ (2016). CXCR4: a key receptor in the crosstalk between tumor cells and their microenvironment. Blood..

[30] Bachelder RE, Wendt MA, Mercurio AM (2002). Vascular endothelial growth factor promotes breast carcinoma invasion in an autocrine manner by regulating the chemokine receptor CXCR4. Cancer Res..

[31] Kitaori T (2009). Stromal cell-derived factor 1/CXCR4 signaling is critical for the recruitment of mesenchymal stem cells to the fracture site during skeletal repair in a mouse model. Arthritis Rheum..

[32] Wei L, Fraser JL, Lu ZY, Hu X, Yu SP (2012). Transplantation of hypoxia preconditioned bone marrow mesenchymal stem cells enhances angiogenesis and neurogenesis after cerebral ischemia in rats. Neurobio. Dis..

[33] Ceradini DJ (2004). Progenitor cell trafficking is regulated by hypoxic gradients through HIF-1 induction of SDF-1. Nat. Med..

[34] Kijowski J (2001). The SDF-1-CXCR4 axis stimulates VEGF secretion and activates integrins but does not affect proliferation and survival in lymphohematopoietic cells. Stem Cells..

[35] Kitaori T (2009). Stromal cell-derived factor1/CXCR4 signaling is critical for the recruitment of mesenchymal stem cells to the fracture site during skeletal repair in a mouse model. Arthritis Rheum..

[36] Jones GN (2012). Upregulating CXCR4 in human fetal mesenchymal stem cells enhances engraftment and bone mechanics in a mouse model of osteogenesis imperfecta. Stem Cells Transl. Med..

[37] Zhu Y, Yao Z, Wu Z, Mei Y, Wu M (2014). Role of tumor necrosis factor alpha‐induced protein 1 in paclitaxel resistance. Oncogene..

