# Supplementary material for: CXCL12 overexpression promotes the angiogenesis potential of periodontal ligament stem cells
Source: Sci Rep. 2017 Aug 31;7:10286. doi: 10.1038/s41598-017-10971-1 (PMC5579269; doi:10.1038/s41598-017-10971-1)
Supplement: Supplementary file 1 — SUPPLEMENTARY INFO [file 41598_2017_10971_MOESM1_ESM.pdf]

CXCL12 overexpression promotes the angiogenesis potential of periodontal ligament stem cells

Lei Zhang[1,2,3], Yong Zhou[2], Xiaoyu Sun [2], Jian Zhou [2], Pishan Yang[1,3]*

1. Department of Periodontology, School of Stomatology, Shandong University, Jinan, Shandong 250012
2. Department of Periodontology, College and Hospital of Stomatology, Anhui Medical University, Hefei 230032, Anhui province, China
3. Shandong Provincial Key Laboratory of Oral Biomedicine, Shandong University, Jinan, Shandong 250012

*Correspondence to Pishan Yang, Ph.D., Department of Periodontology, School of Stomatology, Shandong University, 44-1, West Wen Hua Road, Jinan, Shandong, 250012, P.R. China.

e-mail: yangpishanzl@126.com

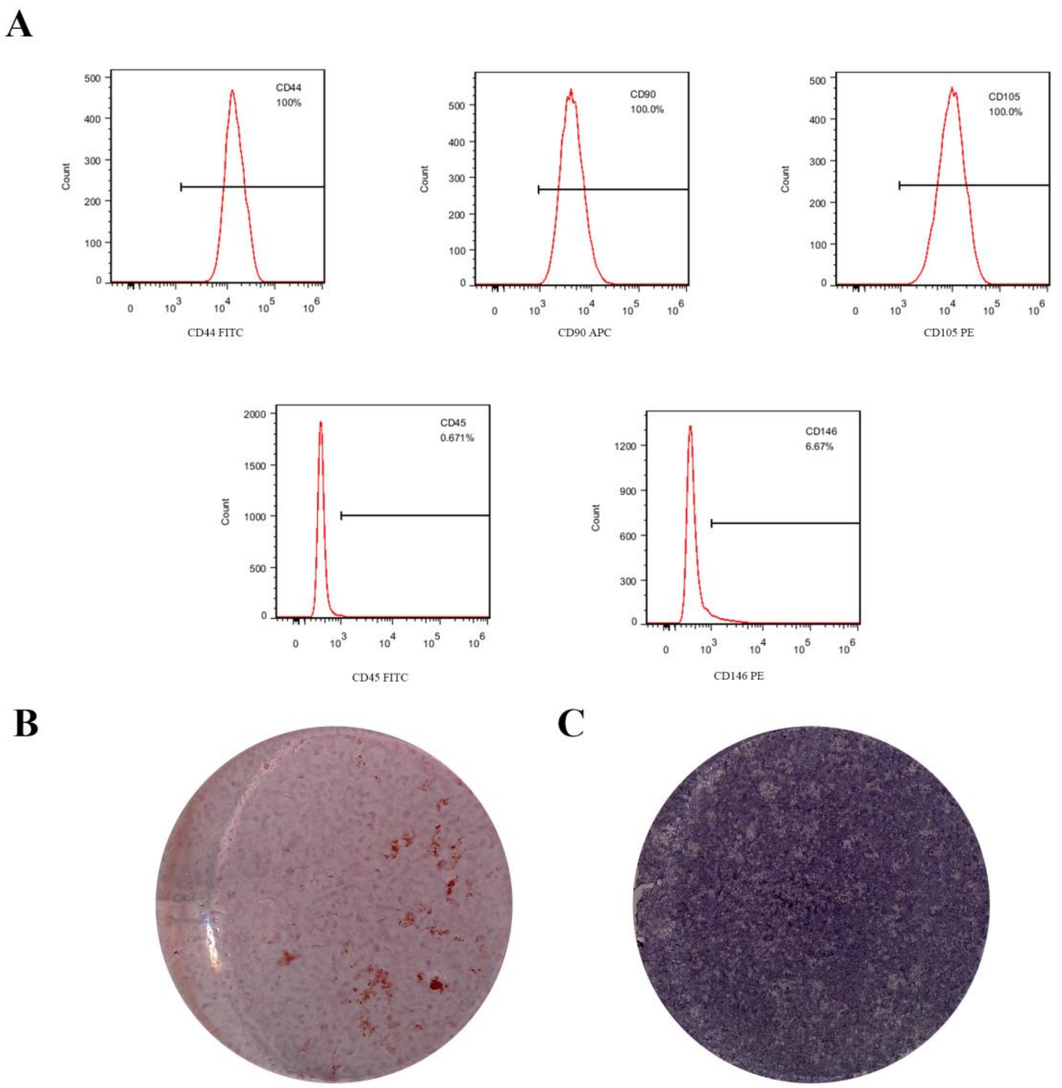

Supporting Fig. S1 PDLSCs' stemness verification. Flow cytometry results showing the MSC marker expression of PDLSCs (A). Oil Red O staining result (B) and alkaline phosphatase (ALP) staining result showing the multi-differentiation potential of PDLSCs.

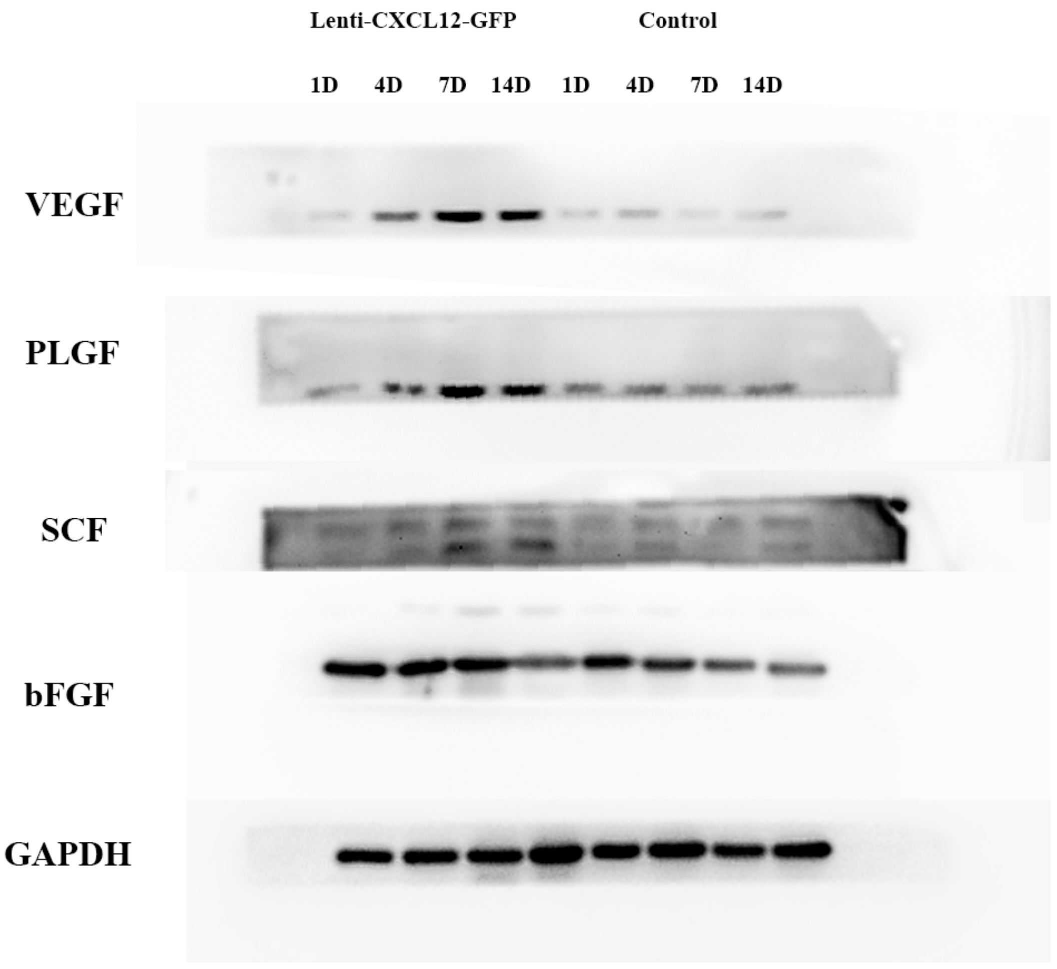

Supporting Fig. S2 Original picture of Western-blot for detecting the expression of VEGF, PLGF, bFGF and SCF.
